# Exploring smooth muscle phenotype and function in a bioreactor model of abdominal aortic aneurysm

**DOI:** 10.1186/1479-5876-11-208

**Published:** 2013-09-12

**Authors:** Kirsten Riches, Timothy G Angelini, Gurprit S Mudhar, Jean Kaye, Emily Clark, Marc A Bailey, Soroush Sohrabi, Sotirios Korossis, Peter G Walker, D Julian A Scott, Karen E Porter

**Affiliations:** 1 Division of Cardiovascular and Diabetes Research, Leeds Institute of Genetics, Health and Therapeutics (LIGHT), University of Leeds, Leeds, UK; 2 Multidisciplinary Cardiovascular Research Centre (MCRC), University of Leeds, Leeds, UK; 3 Department of Cardiothoracic, Transplantation and Vascular Surgery, Hannover Medical School, Hannover, Germany; 4 Institute of Medical and Biological Engineering, School of Mechanical Engineering, University of Leeds, Leeds, UK; 5 School of Biomedical Sciences, Faculty of Biological Sciences, University of Leeds, Leeds, UK; 6 Leeds Vascular Institute, Leeds General Infirmary, Leeds, UK

**Keywords:** Abdominal aortic aneurysm, Smooth muscle cell, Human, Porcine, Bioreactor, Morphology, Proliferation, Senescence, Apoptosis, MMP-2

## Abstract

**Background:**

Vascular smooth muscle cells (SMC) are central to arterial structure and function yet their involvement in the progression of abdominal aortic aneurysm (AAA) disease is not well studied. The progressive and silent nature of AAA in man essentially restricts research to the use of “end-stage” tissue recovered during surgical repair. This study aimed to generate an *ex vivo* model of AAA using protease-treated porcine carotid arteries maintained in a novel bioreactor, and to compare the structural and functional changes in SMC cultured from the recovered vessels with those from human tissue acquired at elective surgical repair.

**Methods:**

Freshly isolated porcine arteries were pretreated with collagenase and/or elastase before culturing under flow in a bioreactor for 12 days. Human end-stage aneurysmal tissue and saphenous veins from age-matched controls were collected from patients undergoing surgery. SMC were cultured and characterised (immunocytochemistry, measurement of spread cell area) and assessed functionally at the level of proliferation (cell-counting) and matrix-metalloproteinase (MMP) secretion (gelatin zymography). Cellular senescence was investigated using β-galactosidase staining and apoptosis was quantified using a fluorescence-based caspase 3 assay.

**Results:**

Co-expression of alpha-smooth muscle actin and smooth muscle myosin heavy chain confirmed all cell populations as SMC. Porcine SMC harvested and cultivated after collagenase/elastase pretreatment displayed a prominent “rhomboid” morphology, increased spread area (32%, P < 0.01), impaired proliferation (47% reduction, P < 0.05), increased senescence (52%, P < 0.001), susceptibility to apoptosis and reduced MMP-2 secretion (60% decrease, P < 0.01) compared with SMC from vehicle, collagenase or elastase pre-treated vessels. Notably, these changes were comparable to those observed in human AAA SMC which were 2.4-fold larger than non-aneurysmal SMC (P < 0.001) and exhibited reduced proliferation (39% reduction, P < 0.001), greater apoptosis (4-fold increase, P < 0.001), and increased senescence (61%, P < 0.05).

**Conclusions:**

Combined collagenase/elastase exposure of porcine artery maintained in a bioreactor under flow conditions induced a SMC phenotype characteristic of those cultured from end-stage AAA specimens. This model has potential and versatility to examine temporal changes in SMC biology and to identify the molecular mechanisms leading to early aberrancies in SMC function. In the longer term this may inform new targets to maintain aortic SMC content and drive cells to a “reparative” phenotype at early stages of the disease.

## Background

Abdominal aortic aneurysm (AAA) rupture carries an ~80% mortality risk and is responsible for 6000 deaths annually in the UK, accounting for 2% of all deaths in men aged >65 years (http://www.aaa.screening.nhs.uk). Once established, AAA progressively evolves towards rupture which is correlated with maximal aneurysm diameter. Intervention by either open surgery or endovascular repair is offered once the annual risk of rupture outweighs the mortality risk associated with intervention [1]. Clinical risk factors for AAA include male gender, age, hypertension, smoking and a family history of aneurysm disease.

The pathology of AAA encompasses infiltration by inflammatory cells (macrophages and lymphocytes), apoptosis of smooth muscle cells (SMC) within the aortic wall, and degradation of the extracellular matrix (ECM) which severely compromises the structural integrity of the vessel rendering it susceptible to rupture [2]. The inflammatory characteristics of AAA have been a major research focus for many years (reviewed in [3]), yet comparatively fewer investigations have considered the role of SMC. Given the inherent plasticity of SMC to remodel vascular walls through acquisition of a dedifferentiated, secretory phenotype [4], this is perhaps surprising.

SMC are the principal resident cells of the aortic wall and are essential in maintaining its structure through controlled proliferation and by secretion and turnover of ECM. Whilst SMC secrete the “building blocks” of ECM (collagens, elastin etc.), they also secrete matrix metalloproteinases (MMPs) that are involved in ECM breakdown [5]. The most extensively characterised with respect to AAA are the gelatinases MMP-2 and MMP-9, both of which are expressed at elevated levels in human and animal AAA tissue specimens [6,7]. Importantly, MMP-2 or MMP-9-deficient mice fail to develop experimental aneurysms [8]. Thus, SMC are capable of maintaining a dynamic ECM that can respond and adapt to the physiological environment [4]. However, during AAA development, inflammatory infiltrates contribute additional proteolytic activity within the ECM and induce SMC apoptosis [5,9], severely compromising vessel tone and structure. SMC within the aortic media are unique in their potential to induce repair in the damaged vessel and this makes them an appealing target for further detailed study.

A major obstacle to AAA research is that human tissue is not available in the early, silent phase of the disease and specimens acquired at the time of surgical repair are likely to have endured cellular and molecular changes over an extended period. A number of studies have elucidated evidence that supports alterations in oxidative stress [10], proliferation [11,12] and MMP-2 activity [11,13] in human AAA-SMC compared to non-aneurysmal SMC. However, by the very nature of the “end-stage” tissue it is not possible to define aberrations in SMC biology that are likely to occur early in disease progression. Murine or rodent models have been generated to facilitate this type of research and include methods that utilise elastase or angiotensin II infusion, or application of calcium chloride to the exposed adventitia of the aorta (reviewed in [14]). These generally result in aneurysm formation within 2-4 weeks. A decellularised guinea pig to rat xenograft model of aneurysm development has also been described [15,16]; however rodent vessel physiology does not mimic human vessels as closely as those from larger animals. An *in vivo* porcine model of infrarenal aneurysm has been investigated [17], and porcine carotid arteries have previously been used *ex vivo* in a bioreactor to study the effect of stent implantation [18]. More recently, an *in vitro* bioreactor model of aneurysm has been described [19] in which PTFE grafts were firstly dilated with a balloon catheter and subsequently seeded with human SMC which over 14 days formed a full “neointima” over the dilated vessel.

The aim of this study was to generate a novel *ex vivo* model of AAA to study the fate, phenotype and function of the SMC specifically. This was undertaken by brief protease exposure of porcine vessels followed by culture under flow conditions in a bioreactor for 12 days. SMC subsequently isolated and cultured from these vessels were then compared with SMC cultured from end-stage human AAA tissue.

## Methods

### Establishing porcine vessels in the bioreactor

**Figure 1:**
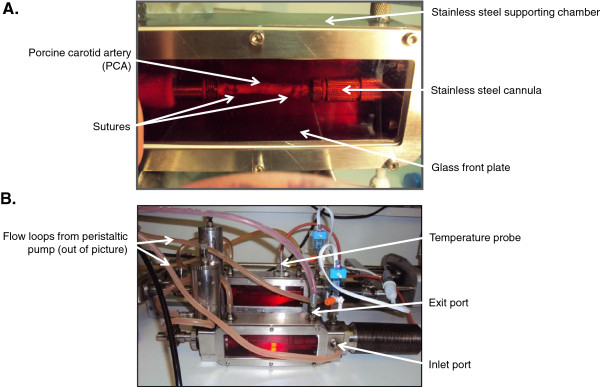
**Bioreactor. (A)** Segments of PCA were secured between stainless steel cannulae. The chamber was assembled by sealing a glass front plate onto the supporting chamber. **(B)** Culture medium was circulated from a reservoir via a peristaltic pump through the inlet cannula to perfuse the interior of the vessel, before leaving through the outflow cannula and perfusing the exterior of the vessel. Media was then returned to the reservoir by flowing through the exit port. Vessels were perfused at constant flow rate for 12 days prior to harvesting for histology and SMC explant culture.

Left and right porcine carotid arteries (PCA) were harvested aseptically from four-month old 65 kg pigs sedated with Stresnil, anaesthetised with Hypnovel and terminated via Pentoject injection. All animal procedures were conducted according to UK Home Office Regulations. Vessels were cleaned of adventitia and superfluous fat, and thin “rings” of vessel (~2-3 mm length) were cut, immediately fixed in formalin and processed for histology. A further tissue fragment was used to prepare SMC from the freshly isolated artery, whilst the remaining vessels were used to prepare two equivalent lengths of artery (each approx 6 cm) which were treated as follows. Ultrapure LMP agarose (Invitrogen) was reconstituted in Hanks balanced salt solution (HBSS, Invitrogen) to form a gel (1% w/v) and this vehicle was applied to control arteries. Enzyme treatments were incorporated into vehicle gel as required (3 mg/ml Type 1A collagenase, 390 U/mg (Sigma-Aldrich); 1.5 mg/ml porcine pancreatic elastase, 50 U/mg (MP Biomedical) or in combination) to the mid-section (~ 2 cm) of the adventitial surface of the vessel using a small brush. Consistency of application was achieved by immobilising the vessels in a sterile dish such that equal volumes (1 ml) of treatment were applied to and retained around this mid-portion during exposure. After a 3 h incubation period at 37°C in a humidified incubator, the vessels were rinsed thoroughly in HBSS and mounted in the bioreactor. In brief, the artery was mounted between two stainless steel cannulae and tied securely with sutures. This was placed inside a stainless steel supporting chamber that was sealed by fixing a custom-made glass plate onto the front long aspect (Figure 1A). Flow was generated using a peristaltic pump which drew culture medium from a primary reservoir before pumping it through a second reservoir in order to eliminate pulsations from the peristaltic pump. Culture medium was delivered via the inlet cannula, flowed through the arterial lumen and exited through the outlet cannula into the chamber. By this means, the exterior of the artery was also continually perfused, with the medium flowing through an exit port and back to the reservoir (see Figure 1B). Each chamber was subjected to identical conditions in separate, steady flow loops of ~120 ml/min using culture medium containing 30% foetal calf serum (FCS) [20] and gassed with 5% CO_2_ in air at 37°C for 12 days.

After the culture interval, vessels were carefully recovered, a section was fixed and prepared for histology and the remaining portions were allocated to SMC culture, ultimately yielding cells derived from vehicle (VEH), collagenase (C), elastase (E) or combination (CCE) treatment groups.

### Histological examination of intact vessels

Formalin-fixed vessels were processed, paraffin-embedded and sectioned to 5 μm. Sections were stained with anti-alpha smooth muscle actin (α-SMA) and Millers elastin as previously described [21]. Images were captured using a Zeiss AxioVision Imaging System (AxioCam HRc camera on an AxioImager Z.1 microscope).

### SMC isolation and culture

AAA tissue (anterior abdominal aortic wall) was obtained from patients undergoing open repair of the infra-renal abdominal aortic aneurysm, and SV fragments obtained from age-and sex-matched patients undergoing coronary artery bypass grafting at Leeds General Infirmary, UK. Local ethical committee permission and informed, written patient consent was obtained, and the study conformed to the principles outlined in the Declaration of Helsinki. Human aortic SMC (1 donor) were purchased from a commercial source. Porcine vessels were used either directly after harvesting (fresh) or upon removal from the bioreactor. From all human and porcine freshly isolated vessels, SMC cultures were established by an explant technique we described previously [22]. Cells were maintained in Dulbecco’s Modified Eagle Medium (DMEM) supplemented with 10% FCS, 1% L-Glutamine and 1% penicillin / streptomycin fungizone (full growth medium; FGM) at 37°C in 5% CO_2_ in air. SMC were serially passaged using trypsin/EDTA as necessary and used for experiments between passages 2-5.

### SMC morphometric analysis

SMC were seeded at a density of 2×10^5^ cells per 75 cm^2^ flask in FGM and cultured for 96 h. Using light microscopy (× 100 magnification) at least 10 fields of view were captured. The cell boundaries of 100 individual cells per experiment/condition were traced and spread cell area was calculated using Image J software (http://imagej.nih.gov/ij/).

### Immunocytochemistry

SMC were seeded at a density of 2×10^3^ in chamber slides, cultured for 4 days in FGM then fixed in 4% paraformaldehyde. Immunostaining for smooth muscle myosin heavy chain (SM-MHC) and α-SMA was performed as we previously described [23]. SMC were visualised using a Zeiss LSM 510 confocal microscope (× 400 magnification).

### Proliferation assays

Proliferation assays were performed as described previously [23]. Briefly, cells were seeded at 1x10^4^ cells per well in 24-well plates, allowed to establish overnight (~18 h) and quiesced in serum-free medium (SFM) for 72 h before performing cell counts in triplicate using trypan blue and a haemocytometer. These counts were designated “day 0”. Cells were then replaced into FGM and further triplicate counts taken on days 2, 4 and 7, with medium being replaced on days 2 and 4. Proliferation curves were plotted and area under the curve (AUC) analysis was performed using GraphPad Prism software (http://www.graphpad.com).

### Apoptosis assay

SMC were plated in 96-well plates at a density of 3×10^3^ cells per well in FGM and established overnight. Cells were treated with 5 μmol/L NucView™ 488 caspase-3 substrate (Biotium) according to manufacturer’s instructions in the absence and presence of 50 nmol/L staurosporine (Sigma-Aldrich). Plates were incubated and imaged using an IncuCyte FLR time-lapse fluorescence microscope (Essen Bioscience) for up to 24 h in phase contrast and fluorescence mode using a ×10 objective, after which all cells were stained using 1 μmol/L Vybrant DyeCycle Green® (Molecular Probes, Invitrogen) and quantified using an inbuilt algorithm to calculate an apoptosis index.

### Senescence-associated β-galactosidase assay

SMC were seeded at 7.5×10^4^ cells per well in 6 well plates and cultured for 48 h in FGM. Cell senescence was quantified using a commercial assay of β-galactosidase (Cell Signaling Technology), according to manufacturer’s instructions. This assay histochemically detects expression of senescence-associated β-galactosidase at pH 6, resulting in a blue precipitate. Ten low power (× 40 mag.) microscopic fields were imaged from each well and a senescence score was calculated.

### Gelatin zymography

SMC were seeded at a density 2×10^5^ cells per 25 cm^2^ flask in FGM, established for 24 h, quiesced in SFM for 72 h, and then treated with medium containing 0.4% FCS or supplemented with phorbol ester 12-O-tetradecanoylphorbol-13-acetate (TPA, 100 nmol/L, Sigma-Aldrich) for a further 48 h. Conditioned medium (CM) was then collected, centrifuged to remove cell debris, snap frozen in liquid nitrogen and stored at −80°C until required. Gelatin zymography of CM was performed as described previously [22].

### Statistical analysis

All data are expressed as mean ± SEM with *n* representing the number of experiments on cells from different patients/animals. Differences between treatment groups were analysed using paired (for porcine) or non-paired (for human) ratio t-tests or repeated measures one-way ANOVA with Newman-Keuls post-hoc tests as appropriate. P < 0.05 was considered statistically significant.

## Results

### Application of collagenase and elastase induces morphological changes in the PCA

**Figure 2:**
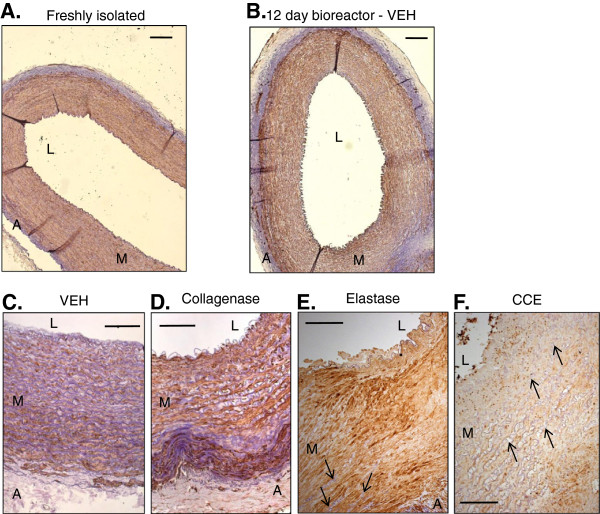
**Histology of PCA.** Tissue was fixed at initial harvest (freshly isolated), and after 12 days culture in the bioreactor following pre-treatment with vehicle control (agarose gel, VEH), collagenase, elastase, or both in combination (CCE). Purple indicates elastin fibres; brown indicates α-SMA-positive regions. L = lumen, M = media, A = adventitia. Low power (× 50 magnification) images of **(A)** freshly isolated PCA and **(B)** VEH, scale bars = 200 μm. Higher power magnification (× 100) of PCA pre-treated with **(C)** VEH, **(D)** collagenase, **(E)** elastase and **(F)** CCE, scale bars = 200 μm. Arrowheads in **(E)** and **(F)** indicate sparse areas of preserved, thinned elastin.

Freshly isolated PCA was compared with VEH treated vessel recovered after 12 days in the bioreactor. Gross appearance of the vessels was comparable and all layers were intact (Figure 2A,B). Conversely, all enzyme-treated vessels displayed variable degrees of degenerative changes in the wall (Figure 2C-F). Histological comparison of PCA pre-treated with VEH (Figure 2C) versus collagenase revealed a loss of smooth muscle integrity (Figure 2D). Vessels treated with elastase alone (Figure 2E) or in combination with collagenase (Figure 2F) also demonstrated a clear loss of elastin fibres.

### Smooth muscle cell phenotype

#### *(i) Porcine carotid arteries*

**Figure 3:**
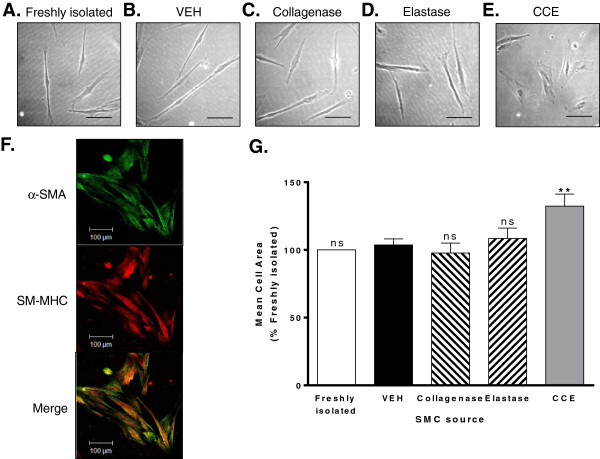
**PCA SMC morphology.** Cells were explanted from both freshly isolated PCA and bioreactor vessels and maintained in cell culture in full growth medium. Representative phase contrast images of cells explanted from **(A)** freshly isolated tissue **(B)**, VEH, **(C)** collagenase, **(D)** elastase and **(E)** CCE-pre-treated vessels, scale bar = 100 μm. **(F)** Immunocytochemical staining for α-SMA (green) and SM-MHC (red) and co-localisation (orange). Magnification × 400, scale bar = 100 μm. **(G)** Mean cell areas of 100 individual cells per condition were quantified using Image J and expressed relative to the matched freshly isolated vessel. All n = 3, ns = non significant, **P < 0.01.

Medial wall cells isolated from both fresh and bioreactor vessels explanted readily in culture, indicative of their viability. Cells propagated from VEH control vessels were indistinguishable from those of fresh vessels and exhibited a characteristic “spindle” appearance of SMC (Figure 3A,B). Despite the observed disruption in whole vessel wall structure, cells cultured from either C-or E-treated arteries were morphologically comparable to VEH (Figure 3C,D). In contrast, those isolated from CCE-treated PCA exhibited a prevalence of flattened, rhomboid cells (Figure 3E). In all cases, cells stained positively for SM-MHC and α-SMA confirming their identity as vascular SMC (Figure 3F).

Quantification of the spread cell areas for each group corresponded with morphological appearances. Whilst cellular areas for fresh, VEH, C and E did not differ, the mean spread area of CCE-SMC was ~ 40% greater than that of VEH-SMC (3720.4 ± 223.2 versus 2707.9 ± 208.5 μm^2^, P < 0.05, Figure 3G).

#### *(ii) Human AAA*

**Figure 4:**
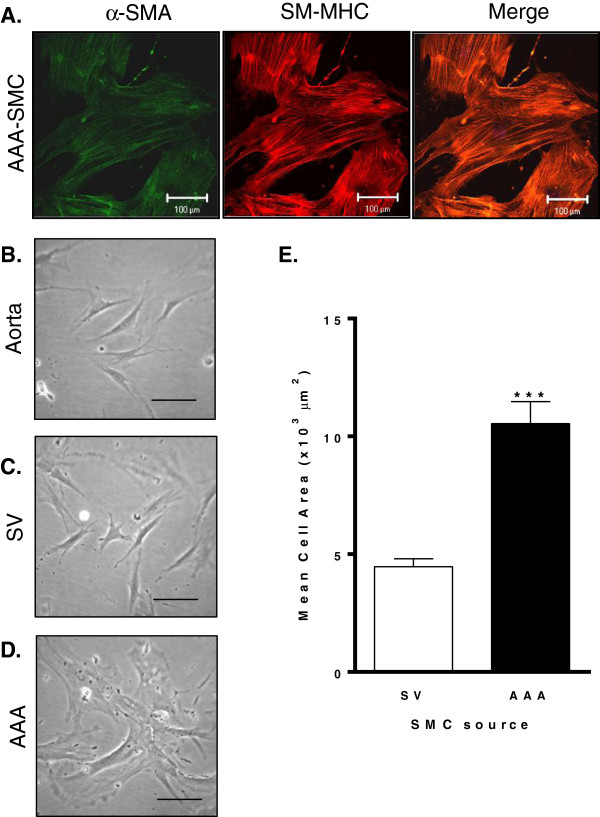
**Human SMC morphology.** Cells were explanted from human aneurysmal wall tissue (AAA), or human saphenous vein (SV) and maintained in culture in full growth medium. **(A)** Immunocytochemical staining of α-SMA (green) and SM-MHC (red) and co-localisation in human AAA SMC. Magnification ×400, scale bar = 100 μm. **(B)** Representative phase contrast images of aortic SMC, **(C)** SV-SMC and **(D)** human AAA-SMC, scale bar = 100 μm. **(E)** The mean cell areas of 100 individual cells per patient were quantified using Image J. All n = 12, ***P < 0.001.

Cells propagated from AAA specimens of 12 different patients were confirmed as SMC by co-expression of α-SMA and SM-MHC (Figure 4A). Morphologically, whilst aortic-SMC and SV-SMC displayed a predominant spindle appearance, AAA-SMC exhibited clear heterogeneity with a predominance of rhomboid cells (Figure 4B-D). The mean cell area of AAA-SMC was 10,537.0 ± 936.6 μm^2^, ~ 2.4-fold larger than SV-SMC (4468.3 ± 335.4 μm^2^, Figure 4E).

### SMC proliferation

**Figure 5:**
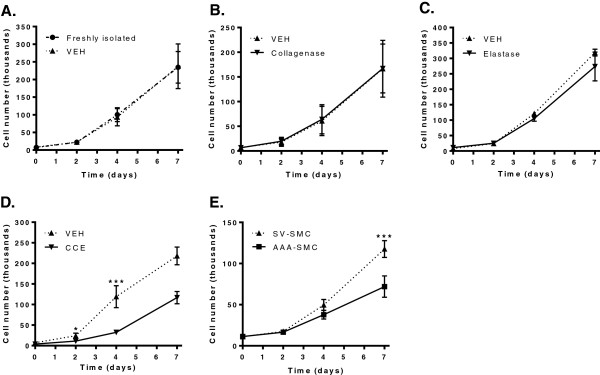
**SMC proliferation.** Cell number was monitored over a 7- day period to generate growth curves in full growth media in the following SMC groups: **(A)** Freshly isolated and VEH (n = 3), **(B)** VEH and collagenase (n = 3), **(C)** VEH and elastase (n = 3), **(D)** VEH and CCE (n = 3), and **(E)** human SV and AAA-SMC (n = 12). *P < 0.05, **P < 0.01, ***P < 0.001.

Porcine SMC proliferation assays were performed over a 7-day interval, over which VEH- SMC and freshly isolated SMC exhibited identical profiles (Figure 5A). Similarly, SMC proliferation from bioreactor vessels with C or E pre-treatment was virtually identical to VEH (Figure 5B,C). However, CCE-SMC showed a significant reduction of ~60% versus VEH (P < 0.001, n = 3; Figure 5D).

AAA-SMC proliferation was compared with non-aneurysmal SV-SMC in a side-by-side manner. Proliferation of AAA-SMC over 7 days was significantly less than that of SV-SMC (Figure 5E), with ~40% reduction in cell number over the period (P < 0.001, n = 12).

### SMC apoptosis

**Figure 6:**
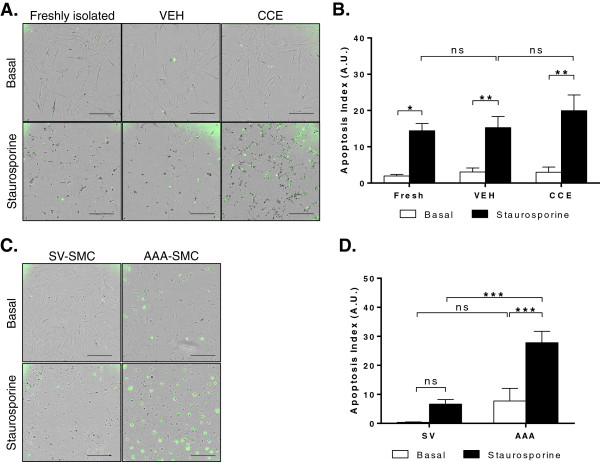
**SMC apoptosis.** Cells were cultured in FGM ±50 nmol/L staurosporine for 24 h. **(A)** Representative images of PCA-SMC (green fluorescence indicates apoptotic cells). Scale bar = 300 μm. **(B)** Quantification of apoptosis index in PCA-SMC (n = 3). **(C)** Representative images of human SV- and AAA-SMC, scale bar = 300 μm. **(D)** Quantification of apoptosis index in human SMC (n = 4). *P < 0.05, **P < 0.01, ***P < 0.001, ns = non-significant.

Apoptosis assays were performed basally and in response to an apoptotic stimulus (staurosporine). All porcine SMC displayed equivalent levels of basal apoptosis that were significantly increased following staurosporine treatment (5-7 fold increase, n = 3, P < 0.05, Figure 6A,B). Whilst CCE-SMC appeared more susceptible to the apoptosis-inducing effect of staurosporine, this increase was not statistically significant (Figure 6A,B).

In human cells, there was a strong trend towards increased basal apoptosis in AAA-SMC compared with SV-SMC (6.6 A.U. vs. 0.4 A.U.) but not statistically significant; there was considerable variability between cell populations (n = 4, Figure 6C,D). However, following a 24 h exposure to staurosporine there was a marked increase in apoptotic cells in the AAA-compared to SV-SMC (27.8 A.U. vs. 6.6 A.U.). Staurosporine-induced apoptosis in SV-SMC was identical to that of AAA-SMC without stimulation (n = 4, P < 0.001, Figure 6C,D).

### SMC senescence

**Figure 7:**
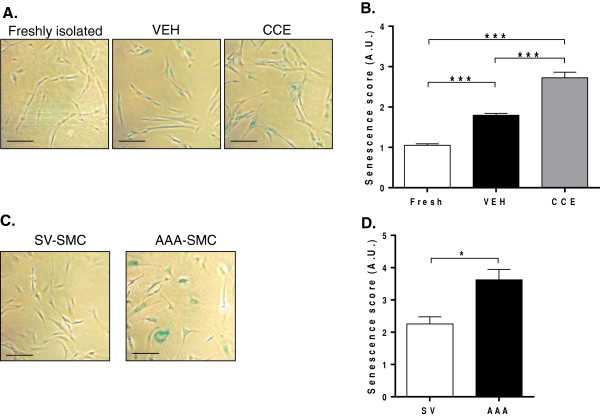
**SMC senescence.** Cells were cultured in FGM for 72 h before senescent cells were detected by β-galactosidase staining at pH 6.0. **(A)** Representative images of PCA-SMC (blue precipitate is observed in senescent cells). Scale bar = 200 μm (n = 3). **(B)** Senescence score in PCA-SMC (n = 3). **(C)** Representative images of human SV-and AAA-SMC, scale bar = 200 μm (n = 4). **(D)** Senescence score in human SMC (n = 4). *P < 0.05, ***P < 0.001.

Cellular senescence was evaluated by measuring expression of β-galactosidase. The incidence of senescent cells in VEH-SMC was higher than in freshly isolated populations (1.79 A.U vs. 1.01 A.U. respectively). However, the extent of senescence in the CCE-SMC was further elevated to 2.72 A.U. (n = 3, P < 0.001 CCE vs. VEH, Figure 7A,B).

Human SV-SMC exhibited a basal level of senescence (2.26 A.U.), and this was significantly higher in AAA-SMC (3.62 A.U., n = 4, P < 0.05, Figure 7C,D).

### Matrix metalloproteinase (MMP) secretion

**Figure 8:**
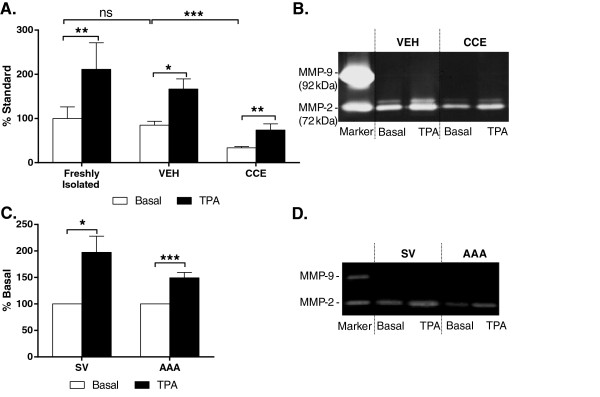
**MMP-2 secretion from SMC.** Equal densities of SMC were cultured in basal media (0.4% FCS) in the presence or absence of 100 nM TPA for 48 h. Conditioned media were analysed for MMP-2 secretion using gelatin zymography. **(A)** Mean densitometry data from SMC from freshly isolated tissue, VEH and CCE-pre-treated vessels (all n = 3, ns = non significant, *P < 0.05, **P < 0.01, **P < 0.001). **(B)** Representative zymogram. **(C)** Mean densitometry data from human SV and AAA-SMC (all n = 12, *P < 0.05, ***P < 0.001). **(D)** Representative zymogram.

All freshly isolated SMC secreted MMP-2 constitutively, regardless of source. In porcine cells, basal secretion of MMP-2 was similar in fresh and VEH cells but was significantly attenuated in CCE-SMC (P < 0.001, n = 3, Figure 8A,B). In all 3 populations, TPA stimulation resulted in ~ 2-fold increase in MMP-2 secretion (fresh P < 0.01; VEH P < 0.05; CCE P < 0.01 versus unstimulated cells) although the absolute levels secreted from the CCE cells were lower than VEH (P < 0.001, n = 3, Figure 8A,B). Consistent with porcine cells, human SMC secreted MMP-2 basally and this was further increased by TPA stimulation in both SV (2-fold, P < 0.05) and AAA (1.5-fold, P < 0.001; Figure 8C,D).

MMP-9 secretion was not detected under any condition in porcine or human SMC, either basally or with TPA stimulation.

## Discussion

This study has revealed a number of key findings. Firstly we maintained viable porcine carotid arteries under flow conditions in a bioreactor model for 12 days. Histological examination revealed that vessel wall architecture in control (VEH) vessels was identical to that of freshly isolated PCA, but protease-pre-treatments either individually or combined, led to visible disruption of the arterial wall. Within the time frame studied and under these conditions we did not however, observe an unambiguous dilatation of the vessel although we speculate that the thinning we observed preceded overt dilation which may well become apparent at a later time point. Secondly, viable cells were cultured from all vessels and confirmed as SMC through co-expression of α-SMA and SM-MHC. All porcine SMC exhibited characteristic spindle morphology with the exception of those cultured from the combined protease treated vessels that were more rhomboid, a trait common to dedifferentiated, often pathological SMC [24]. The aberrancies in PCA-SMC morphology evident after treatment with CCE were recapitulated in SMC from end-stage human AAA tissue. In agreement with a previous report, AAA-SMC were morphologically distinct (large and rhomboid) from SV-SMC (smaller, spindle) [12] and also from the aortic SMC (spindle) obtained from a commercial source.

SMC phenotypic switching underlies their unique ability to elicit compensatory responses to vascular injury. Indeed, increased SMC proliferation is a prominent feature of occlusive vascular diseases [25]. Conversely, it is well established that SMC depletion is a hallmark of AAA [26,27], which might suggest functional inability of the SMC to remodel the degenerating aortic wall. In this study we revealed that AAA-SMC consistently proliferated more slowly than non-aneurysmal SV-SMC cultured from age- and sex-matched patients. Similarly, the proliferative capacity of SMC was reduced to a similar degree in porcine CCE-SMC compared with paired VEH cells. Reports relating to proliferative capacity of AAA-SMC compared to non-aneurysmal SMC are at variance; claims of both increased [11,28] and decreased proliferation [12] have been documented. In the latter, AAA-SMC consistently proliferated by up to 70% less than inferior mesenteric artery SMC [12], comparator cells that were cultured from the same patients. In the current study we examined SMC from AAA and SV sources from a total of 24 different patients. Given our expertise and familiarity with inherent variability between individual patients [23,29,30] and our documented evidence supporting the intrinsic heterogeneity of SMC populations [31], this is an important aspect of the current study. Whilst SMC derived from age-matched non-aneurysmal abdominal aorta may be a superior comparator, in the current study this was not a feasible option and thus a limitation.

Loss of aortic SMC through apoptosis is a prominent feature of AAA disease [5,27]. The similarity in basal apoptosis we observed between aneurysmal and non-aneurysmal SMC concurs with a previous report where no differences were observed between AAA and matched inferior mesenteric artery SMC cultured under standard conditions [12]. However, we noted significantly augmented apoptosis in AAA-SMC upon exposure to staurosporine. It is conceivable that in AAA disease, SMC apoptosis *in vivo* may be attributable to a heightened sensitivity to apoptotic stimuli in a significant proinflammatory environment, rather than a difference in basal apoptosis levels.

Accelerated vascular aging, cell senescence and synthetic SMC phenotypes have been documented in AAA patients or those with risk factors for AAA [11,12,32,33]. Another common feature of aged cells is that of telomere shortening and this has been demonstrated in both AAA-SMC [10] and leucocytes of patients with AAA [34]. Rhomboid SMC are more commonly reported in pathological states [4] and there is speculation that aging causes a general switch towards a synthetic phenotype in vascular SMC [35]. Aging has been demonstrated to alter SMC proliferation in a variety of ways depending on source and model, and also to modulate the proliferative response to growth factors or cytokines [35]. In keeping with this concept, we also noted differential senescence between PCA vehicle-treated and CCE-SMC, and likewise between human SV and AAA-SMC. It is reasonable to suggest that the SMC phenotypes we identify in both the human AAA and porcine CCE are indicative of accelerated aging.

Another defining feature of end-stage AAA disease is breakdown of the ECM, with marked degradation of elastin fibres [2]. In addition, collagenase activity is elevated in AAA tissue [36,37]. Evidence from pathological specimens suggests that loss of elastin is an early event mediated by SMC [38] and is associated with production of MMP-2 from SMC themselves [13,39]. Elevated expression levels of both MMP-2 mRNA and protein have been reported in human and animal AAA tissue [6,7]. The observed deficiencies in PCA-SMC morphology and proliferation after CCE treatment were also evident at the level of MMP-2 secretion in which we observed, contrary to previous reports, that both basal (constitutive) and phorbol ester-stimulated secretion of MMP-2 from CCE-SMC was significantly lower than from VEH-SMC. The unpaired nature of AAA-SMC and SV-SMC precluded a direct comparison between them although we noted that absolute levels of MMP-2 secretion from AAA-SMC were consistently lower than from equivalent densities of SV-SMC under identical conditions. Interestingly, a study using tissue biopsies from the UK Small Aneurysm Trial concluded that MMP-2 may only play an “etiopathogenic” role in small (<5.5 cm) aneurysms [40] and moreover, significant quantities were bound to the ECM [7]. MMP-2 may actually provoke aneurysm formation rather than propagate their growth, a concept that could only be verified by conducting studies early in the disease process. The availability of human early-stage AAA tissue is however, scarce, primarily because there is insufficient evidence to recommend surgical intervention for small (<5.5. cm) AAA [1,41]. In the present study we found no evidence of MMP-9 secretion from either human or porcine SMC. Whilst MMP-9 levels were associated with AAA rupture in one study [42], in another they were not [43].

To elucidate the function and fate of SMC in the pathogenesis of AAA in man would necessitate access to aortic tissues at all stages of the disease, from initiation through progression to end stage. Since this is not possible, the need for appropriate laboratory models is evident. Whilst large animal models have chiefly been employed to test endovascular stent devices, rodent models have been useful in elucidating molecular mechanisms to identify new treatment options, all of which have employed a range of techniques to induce the experimental aneurysms (reviewed in [14]). Two consecutive published studies support the concept that preservation of vascular SMC content and functionality can limit early aneurysm development. In the first, de-cellularised guinea pig aortic scaffolds were implanted into rats and immediately infused with syngeneic rat SMC. After 8 weeks, vessel expansion was diminished in the SMC-populated vessels and the authors concluded that SMC conferred a protective effect on the graft wall via a paracrine mechanism [15]. Conversely, absence of SMC led to greater dilatation, indicating that SMC perform important roles early in aneurysm formation by protecting against inflammation and proteolysis. A later, similar study by the same investigators introduced SMC to the graft 2 weeks after implantation in order to explore the effect of restoring SMC function in a developing aneurysm. In that study, SMC formed an intima over the top of accumulated thrombus that appeared to stabilise the wall and prevent further dilatation [16].

Of the animal models, porcine arterial vessels exhibit a similar structure to man [14]. An *in vivo* porcine model has also been previously generated by aortic perfusion of a combination of collagenase and elastase to generate an aneurysm [17]. Whilst such large models are valuable, their size and cost implications are substantial, such that time-course studies examining progression of AAA from the early stages and beyond are routinely prohibitive. Our study endorses the need for a robust *ex vivo* model that is amenable to temporal study of SMC dysfunction. After 12 days in the bioreactor, we observed that porcine CCE-SMC appeared phenotypically and functionally similar to SMC cultured from human “end-stage” tissue. The design of our model is conducive to sequential examination of SMC characteristics at earlier time points at which changes in SMC phenotype may be detectable. SMC phenotypic modulation has been demonstrated in a mouse model of AAA and associated with changes in gene expression that are apparent well before aneurysm formation is detected [6]. Given the implementation of national screening programmes for AAA [44,45] it is likely that in the future, diagnosis can be made much earlier in the natural history of the disease. Importantly, such patients are those in whom medical therapy may be pertinent by way of preserving SMC integrity and function through targeting them to a “reparative” phenotype.

## Conclusions

Loss of arterial wall structure and integrity by impaired SMC function provides an explanation for the substantial and progressive weakening of the aortic wall observed in AAA. In order to understand early changes in SMC behaviour, an *ex vivo* model is appropriate and here we have shown that enzyme pre-treatment of porcine carotid arteries maintained for 12 days within a bioreactor generates vessel wall disruption and SMC aberrancies comparable to those of end stage human tissue. Future studies with this engineered bioreactor will allow control of the physical environment experienced by the cultured tissues and thus it holds significant potential for studying SMC dysfunction throughout early aneurysm development. Identifying key cellular and molecular mechanisms that promote SMC loss and aneurysm expansion will inform new therapeutics to preserve SMC content and integrity in the aortic wall.

## Abbreviations

α-SMA: Alpha smooth muscle actin; AAA: Abdominal aortic aneurysm; A.U: Arbitrary units; AUC: Area under curve; C: Collagenase treatment group; CCE: Combined collagenase and elastase treatment group; CM: Conditioned medium; DMEM: Dulbecco’s Modified Eagle Medium; E: Elastase treatment group; ECM: Extracellular matrix; FCS: Foetal calf serum; FGM: Full growth medium; MMP: Matrix metalloproteinase; PCA: Porcine carotid artery; SFM: Serum-free medium; SM-MHC: Smooth muscle myosin heavy chain; SMC: Smooth muscle cell; TPA: 12-O-tetradecanoylphorbol-13-acetate; VEH: Vehicle treatment group.

## Competing interests

The authors declared that they have no competing interests.

## Authors’ contributions

KR led and participated in execution of porcine and human cellular studies and helped draft the manuscript. TGA performed human cell studies and GSM performed porcine cell studies. SK and PGW designed and developed the bioreactor. JK and KEP established and ran the bioreactor experiments. JK and EC conducted the porcine immunohistochemical studies. MAB designed and performed the apoptosis studies. SS was responsible for all aspects of porcine surgery. DJAS obtained funding, provided clinical perspective and gave critical intellectual input. KEP conceived and managed the study, had overall responsibility for its execution and critically revised the manuscript for submission. All authors read and approved the final manuscript.
